# The Case of the Young Male with a Longstanding History of Dyspeptic Symptoms: Peritoneal Tuberculosis

**DOI:** 10.7759/cureus.29612

**Published:** 2022-09-26

**Authors:** Ayesha Iftikhar, Maryam Nisar, Hassan S Sheikh, Faisal Sultan

**Affiliations:** 1 Department of Internal Medicine, Shaukat Khanum Memorial Cancer Hospital & Research Centre, Lahore, PAK; 2 Department of Pathology, Shaukat Khanum Memorial Cancer Hospital & Research Centre, Lahore, PAK; 3 Department of Medical Oncology, Shaukat Khanum Memorial Cancer Hospital & Research Centre, Lahore, PAK

**Keywords:** tuberculosis, abdominal tb, peritoneal tb, dyspepsia, peritoneal tuberculosis, portal vein thrombosis

## Abstract

Portal vein thrombosis associated with peritoneal tuberculosis is an uncommon manifestation of extrapulmonary tuberculosis. We report one such case of a 33-year-old male with a one-year history of dyspepsia, having been on proton pump inhibitors all this time with temporary relief. In view of ongoing symptoms, an endoscopy was done, which at first showed duodenal ulcer. On repeat endoscopy after an interval, there was evidence of mucosa-associated lymphoid tissue (MALT) lymphoma, which prompted a host of investigations in the patient. A positron emission tomography (PET) scan revealed extensive omento-peritoneal involvement along with a hypodense lesion in the liver with interval development of portal vein thrombosis on a CT scan of the abdomen. The biopsy of the hepatic lesion showed granulomatous inflammation. Faced with a diagnostic dilemma, finally, a laparoscopic biopsy was done, which confirmed the diagnosis of peritoneal TB with portal vein thrombosis.

This case highlights the importance of keeping a high index of suspicion to include tuberculosis as a differential when presented with a case such as this and to conduct appropriate investigations to establish the correct diagnosis.

## Introduction

Tuberculosis (TB) is still among the top 10 causes of death worldwide, second only to coronavirus disease 2019 (COVID-19), with a particularly high burden in developing countries [1]. According to a study conducted in Pakistan in 2016, the incidence of abdominal TB (including peritoneal, mesenteric lymphadenitis, and GI tract) out of a total of 54,092 tuberculosis cases was 21% [2]. It is a curable disease if diagnosed in a timely manner, making it imperative to be aware of the various uncommon manifestations and their associations to be able to initiate appropriate treatment. A systematic review reported human immunodeficiency virus (HIV) infection, alcoholic liver disease (ALD), advanced renal disease, and peritoneal dialysis as the commonly observed risk factors of peritoneal TB [3]. Another important predisposing factor is the use of immunosuppressant drugs such as tumor necrosis factor-alpha (TNF-α) inhibitors, thus requiring the exclusion of latent TB infection prior to treatment [4].

Portal vein thrombosis occurring in association with peritoneal TB is an unusual entity that has been reported in only a few case reports. It can easily mimic intra-abdominal malignancy clinically, including ovarian carcinoma and carcinomatosis peritonii, presenting a diagnostic dilemma [5]. A case series of 10 patients suspected of having advanced ovarian tumor, with raised serum CA-125 level, and radiological findings of ascites, peritoneal thickening, and nodules over adnexa, were diagnosed with peritoneal TB following laparotomy [6]. The delays in diagnosis can significantly contribute to increased mortality [7].

## Case presentation

A 33-year-old male, with no comorbid conditions, presented with ongoing dyspepsia for a year for which he had been on proton pump inhibitors with only temporary relief of symptoms. There was no associated abdominal pain, nausea, vomiting, altered bowels, weight loss, fever, or any other systemic complaints. Physical examination was unremarkable except for slight pallor. Initial workup was suggestive of iron-deficiency anemia with hemoglobin of 9.1 g/dl and mean corpuscular volume (MCV) of 58.2 fl. Serum iron was 22 ug/dL with total iron-binding capacity (TIBC) of 303 ug/dL and transferrin saturation of 7.26 %. Fecal occult blood was negative. *Heliobacter pylori *antigen in stool was checked keeping in view his dyspeptic symptoms, which turned out to be negative. He continued to have symptoms of retrosternal burning despite the use of gastric protective agents, which prompted a host of investigations. Based on his symptoms, the differential diagnosis considered included *H. pylori *gastritis, gastroesophageal reflux disease, and granulomatous gastritis.

**Figure 1:**
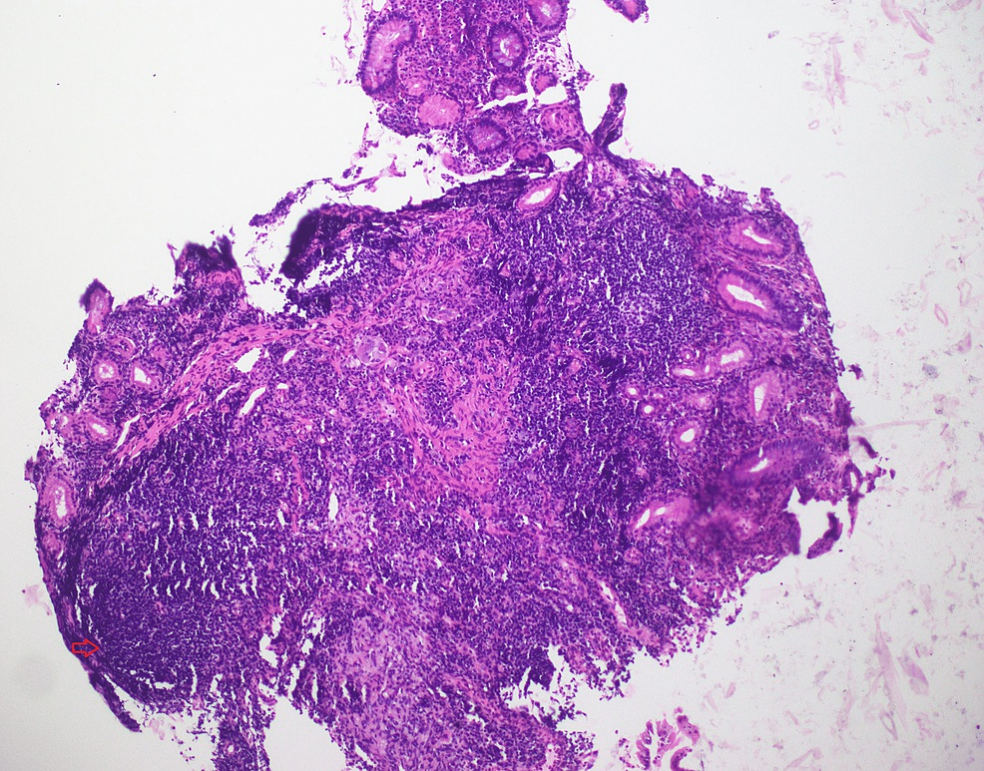
Duodenal mucosa showing atypical lymphoid infiltrate replacing the glands

Upper GI endoscopy showed a significant oozing ulcer at the superior duodenal angle with ulcerated mucosa and underlying abscess on histopathology while colonoscopy was unremarkable. Given his continued symptoms, upper GI endoscopy was repeated at an interval of two months, which showed narrowing at the superior duodenal angle, not allowing onward passage of scope. Histopathological examination of the duodenal lesion revealed features of low-grade B-cell non-Hodgkin lymphoma consistent with mucosa-associated lymphoid tissue (MALT) lymphoma (Figure 1).

**Figure 2:**
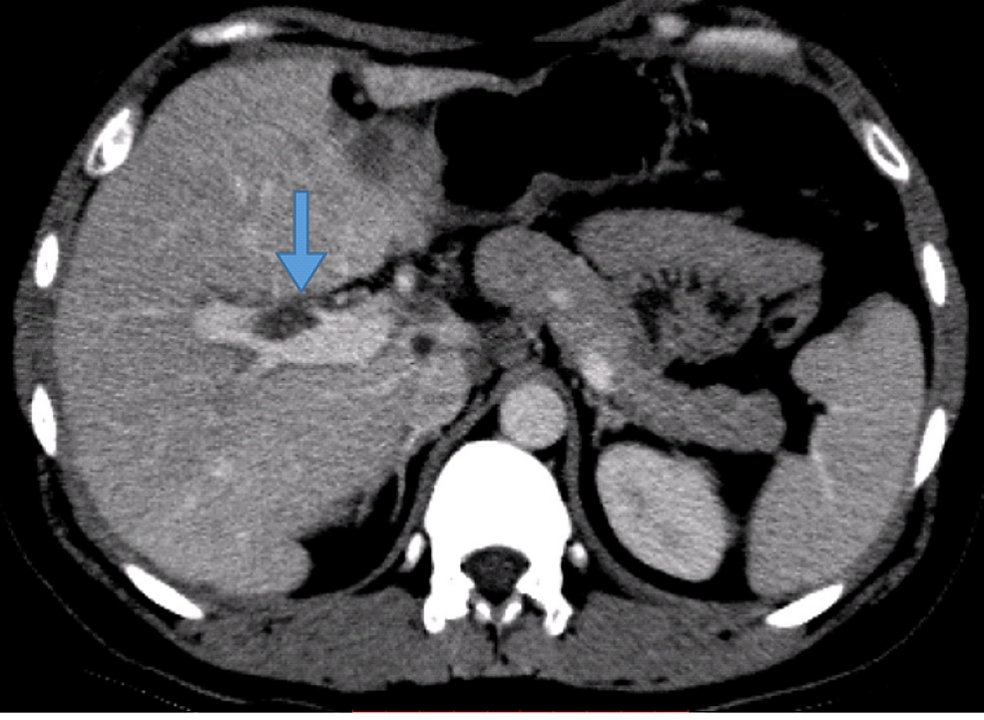
Portal vein thrombus

This led to a positron emission tomography (PET)/CT scan, which showed diffuse circumferential thickening involving gastric pylorus and the first and second parts of the duodenum as well as a hypodense lesion of 3.6 cm in segment III/IV of the liver with multiple enlarged celiac axis, para-aortic, aortocaval, mesenteric and pelvic side wall lymph nodes. A core biopsy of suspicious liver lesion was done for confirmation of distant spread of the disease; however, it only showed mild inflammation and steatosis with no evidence of lymphoma. A bone marrow biopsy was performed as part of the lymphoma workup and it showed normal trilineage hematopoiesis with no evidence of lymphomatous involvement. Considering a sampling error, a core liver biopsy was repeated and it was suggestive of granulomatous inflammation but was negative for Ziehl Neilson stain. The case was discussed in the multidisciplinary tumor board and it was decided to repeat imaging studies and perform an excisional biopsy of the abdominoperitoneal lesions seen on the PET scan. A CT scan abdomen and pelvis was performed and it again showed progression of diffuse peritoneal and omental deposits along with fat stranding and a new finding of portal vein thrombosis (Figure 2). The workup for underlying coagulopathy turned out to be negative.

**Figure 3:**
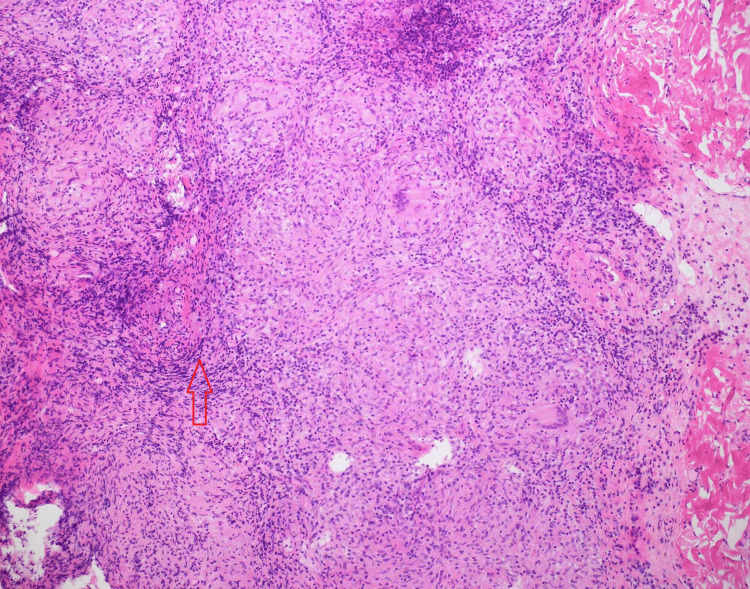
Granulomatous inflammation involving the omentum with epithelioid cell collections forming granulomas and langerhan type giant cells

Finally, a diagnostic laparoscopy was undertaken. Perioperative findings included omento-peritoneal nodules as well as nodules over the small and large bowel and the inferior surface of the liver. The omentum was adherent to the anterior abdominal wall and moderate ascites was noted. Excisional peritoneal nodule biopsy revealed chronic granulomatous inflammation with focal necrosis (Figure 3).

The culture of the biopsy specimen turned out to be positive for *Mycobacterium tuberculosis*, which was pansensitive while the ascitic fluid culture for *Mycobacterium tuberculosis* was negative. During the course of the workup, HIV serology turned out negative.

The patient was started on anti-TB therapy consisting of isoniazid, rifampicin, ethambutol, and pyrazinamide. After completing two months of intensive phase, he was switched to isoniazid and rifampicin for the next four months. He is currently on our follow-up and is doing well with the resolution of dyspepsia. Follow-up endoscopy showed marked resolution of the duodenal angle narrowing. The final diagnosis was omento-peritoneal TB with portal vein thrombosis.

## Discussion

The peritoneum is an uncommon site of extrapulmonary TB with more commonly involved sites being the lymph nodes and pleura with GI involvement constituting about 3.5% of the cases [8]. It occurs either due to spread from an adjacent diseased focus, active pulmonary disease, or from reactivation of latent disease established as a result of hematogenous dissemination [8,9]. Peritoneal TB most commonly presents with abdominal pain (31-94%) and fever (45-100%) [9]. According to a study conducted in Korea, risk factors of peritoneal TB included chronic hepatitis B, alcoholism, end-stage renal disease (ESRD), diabetes, and malignancy [10].

The most common manifestation of peritoneal TB is the exudative variety [11] with ascitic fluid protein content of more than 3g/dl and a total cell count of 150-4000/μL with lymphocyte predominance [12]. The yield of two of the most commonly used tests to establish the diagnosis of peritoneal tuberculosis, i.e; ascitic fluid for Ziehl Neilsen smear and culture, is quite low with a reported sensitivity of only 0-6% [13] and 20% [14], respectively, with an added disadvantage of a long incubation period, further delaying the diagnosis, which has been reported to be associated with increased mortality [7]. According to a retrospective study conducted over a span of seven years in Qatar, Ziehl Neilsen stain positivity was reported at 37% [15]. A non-invasive test with a quick turnaround time that can be useful is ascitic fluid adenosine deaminase level (ADA) using a cut-off level above 36U/L approaching sensitivity and specificity of 100% and 97%, respectively [16,17]. However, the gold standard remains peritoneal biopsy, either open or laparoscopic, for cultures and histopathological examination [7].

Portal vein thrombosis occurring as a complication, especially in an otherwise healthy individual without any comorbid conditions, is a rare presentation described in only a few case reports [18-20]. In one of the reported cases, a 43-year-old male suffered from progressive weight loss over two years and was found to have a mesenteric mass confirmed to be tuberculous on tissue culture [18]. In another instance, a young female with progressive weight loss and abdominal distension with radiologic findings of peritoneal nodules and portal vein thrombosis turned out to have peritoneal TB after a peritoneal biopsy. Contrary to the abovementioned cases, our patient had symptoms of dyspepsia only [20]. 

## Conclusions

A few important points that need to be highlighted in our case include the atypical presentation of peritoneal tuberculosis, in terms of symptoms, complicated by portal vein thrombosis in an immunocompetent patient in terms of symptoms and the diagnostic challenges encountered. This underscores the need to have a high index of suspicion to consider this diagnosis, particularly in high TB burden settings. 
